# Metallothionein-3-mediated intracellular zinc mediates antioxidant and anti-inflammatory responses in the complete Freund’s adjuvant-induced inflammatory pain mouse model

**DOI:** 10.1038/s41420-025-02322-1

**Published:** 2025-02-04

**Authors:** Ngoc Buu Tran, Sook-Jeong Lee

**Affiliations:** Department of Bioactive Material Sciences and Research Center of Bioactive Materials, Jeonbuk National University, Jeonju, Jeonbuk-do 54896 Republic of Korea

**Keywords:** Cell biology, Rheumatoid arthritis

## Abstract

Chronic inflammatory pain is often caused by peripheral tissue damage and persistent inflammation. This disease substantially affects patients’ physical and social well-being. We investigated the role of *metallothionein-3* (*MT3)* in modulating complete Freund’s adjuvant (CFA)-induced intracellular Zn^2+^ activity in an *MT3* knockout mouse model of inflammatory pain in the hind paw. The results demonstrated that increasing intracellular Zn^2+^ levels ameliorate deficits in motor behavior, as well as inflammation in the paw, spleen, and thymus. Furthermore, intracellular Zn^2+^ was crucial in regulating oxidative stress markers (glutathione, superoxide dismutase, catalase, and malondialdehyde) and inflammatory cytokines, such as tumor necrosis factor-α and interleukin-6, in *MT3* knockout mice induced with CFA. This study highlights the critical role of *MT3* in coordinating the intracellular interaction with Zn^2+^, which is vital for the immune systems’s protective functions. These interactions are fundamental for maintaining metal ion homeostasis and regulating the synthesis of various biomolecules in the body.

## Introduction

Inflammatory pain is a clinically prevalent condition characterized by persistent nociceptive hypersensitivities, including a marked reduction in pain thresholds (allodynia) and amplified responses to noxious stimuli at injury sites and surrounding tissues (hyperalgesia). Chronic inflammatory pain often arises from peripheral tissue damage and sustained inflammation [1–4]. Such pain can result from thermal, chemical, or mechanical injuries that activate nociceptors within the nervous system [5]. These inflammation-induced changes typically lead to hypersensitization of the chemical environment surrounding nerve fibers [5].

One of the most common inflammatory pain is arthritis pain, particularly joint pain, which serves as a major trigger for the chronic condition rheumatoid arthritis (RA) [6]. Current therapeutic approaches, including antidepressants, anticonvulsants, and cyclooxygenase inhibitors, are frequently used; however, their efficacy is often accompanied by adverse side effects [7]. Despite extensive research, the molecular and cellular mechanisms underlying the development and persistence of inflammation-induced pain remain poorly understood, posing significant challenges for the identification of novel therapeutic strategies [8].

Symptoms of inflammatory pain are frequently linked to localized immune cell accumulation, stromal expansion, and vascular inflammation, along with the release of proinflammatory cytokines from immune cells. Key cytokines implicated in inflammatory pain pathogenesis include tumor necrosis factor-alpha (TNF-α), interleukin (IL)-1, IL-6, IL-8, etc, … [9, 10]. Advances in the understanding of inflammatory pain in the paw suggest that a cytokine-driven inflammatory cascade underpins joint inflammation and associated pain [11]. Consequently, inflammatory pain treatment strategies have focused on curbing the release of inflammatory mediators. Oxidative stress also plays a pivotal role in the pathophysiology of inflammatory pain in the paw by contributing to joint destruction through reactive oxygen species-mediated lipid peroxidation and impaired antioxidant defense systems, including enzymes such as catalase (CAT), superoxide dismutase (SOD), and glutathione (GSH) [12]. Oxidative stress not only drives tissue damage, but also amplifies inflammatory signaling within cells and tissues [13].

Bones serve as vital zinc reservoirs and contain ~30% of the body’s zinc [14]. Although the regulation of systemic zinc homeostasis is not well-understood, over 30 proteins that control cellular zinc levels have been identified [15, 16]. Cytosolic zinc, an integral structural and catalytic component of various proteins, plays an essential role in numerous cellular responses [16, 17]. Zinc also functions as a signaling molecule in the nervous and immune systems [18, 19]. The intracellular concentration of free zinc (Zn^2+^) is primarily regulated by metallothionein (MT) and zinc transporters [15, 20].

MTs are cysteine-rich proteins that bind to and regulate cellular transition metals and modulate metal toxicity, free radical scavenging, and metal metabolism in response to stimuli such as heavy metals, oxidative stress, and inflammation. The MT family includes four isoforms: MT1, MT2, MT3, and MT4. Although MT1 and MT2 are widely expressed, MT3 is primarily located in the brain [21–23]. MT1/2 may play a role in inflammatory arthritis, with reduced expression linked to symptom alleviation in a collagen-induced arthritis model [24]. MT3, distinguished by its unique structure and strong hydroxyl radical scavenging ability, differs substantially from other MTs in its response to ionic metals [25–27].

The complete Freund’s adjuvant (CFA)-induced inflammatory pain mouse model is one of the models widely used in arthritis research to investigate the pathophysiological mechanisms of this disease [28–31]. This study was conducted to evaluate the role of MT3 in the regulation of intracellular Zn^2+^ activity and its effects on inflammation, oxidative stress, and cytokine levels in CFA-induced inflammatory pain using an *MT3* knockout mouse model. Specifically, we assessed the protective effects of MT3 against oxidative stress and cytokine-mediated inflammatory pain, focusing on its association with intracellular Zn^2+^ activity.

## Results

### Intracellular Zn^2+^ and *MT3* modulate paw swelling and arthritis scores in a CFA-induced inflammatory pain in *MT3* knockout mouse model

**Fig. 1:**
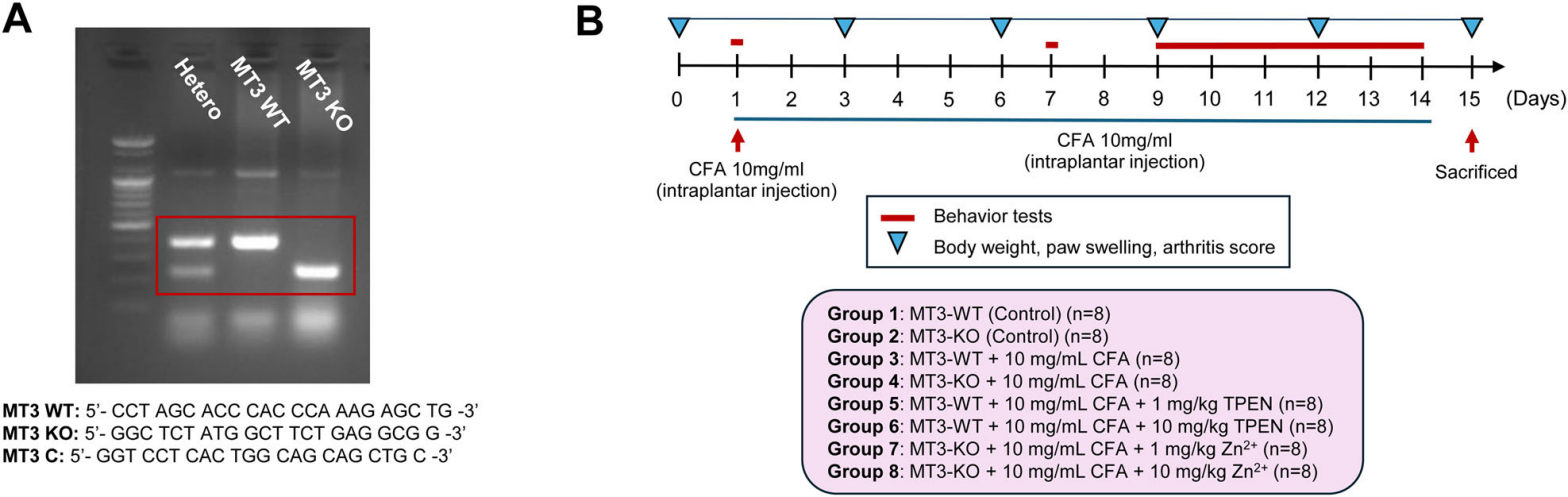
Genotyping and schematic diagram of drug treatment in complete Freund’s adjuvant (CFA)-induced inflammatory pain in mice. **A** Genotyping analysis of *MT3* to identify three genotypes: *MT3* wild-type (WT), *MT3* knockout (KO), and *MT3* heterozygous (Hetero). **B** Behavioral assessments of CFA-induced inflammatory pain mouse model. Behavioral tests were conducted from day 9 (D9) to day 14 (D14), with experimental animals sacrificed on day 15 (D15). The mice were divided into eight experimental groups as follows: (1) *MT3* WT (control group) (*n* = 8) received 0.9% saline and 2% dimethyl sulfoxide (DMSO), (2) *MT3* KO (control group) (*n* = 8), (3) *MT3* WT + 10 mg/mL CFA (*n* = 8), (4) *MT3* KO + 10 mg/mL CFA (*n* = 8), (5) *MT3* WT + 10 mg/mL CFA + 1 mg/kg TPEN (*n* = 8), (6) *MT3* WT + 10 mg/mL CFA + 10 mg/kg TPEN (*n* = 8), (7) *MT3* KO + 10 mg/mL CFA + 1 mg/kg ZnCl_2_ (*n* = 8), and (8) *MT3*-KO + 10 mg/mL CFA + 10 mg/kg ZnCl_2_ (*n* = 8). Results are presented as the mean (*n* = 8) ± SD. Statistical significance: *P < 0.05, **P < 0.01, ***P < 0.001 compared to the control group; ^#^*P* < 0.05, ^##^*P* < 0.01, ^###^*P* < 0.001 compared to CFA-treated group; *ns*, not significant.

*MT3* wild-type (WT) and knockout (KO) mice were generated by crossbreeding *MT3* heterozygous mice, as described in the Methods section. Based on the genotyping results illustrated in Fig. 1A, both *MT3* WT and KO mice were selected. The experiment spanned 15 days (Fig. 1B). The mice were divided into eight groups, each consisting of eight animals, and administered various drug combinations from days 1 to 14, after which the mice were sacrificed for further analysis (Fig. 1B). Behavioral assessments were conducted on days 1, 7, and 9 through 14 (Fig. 1B).

**Fig. 2:**
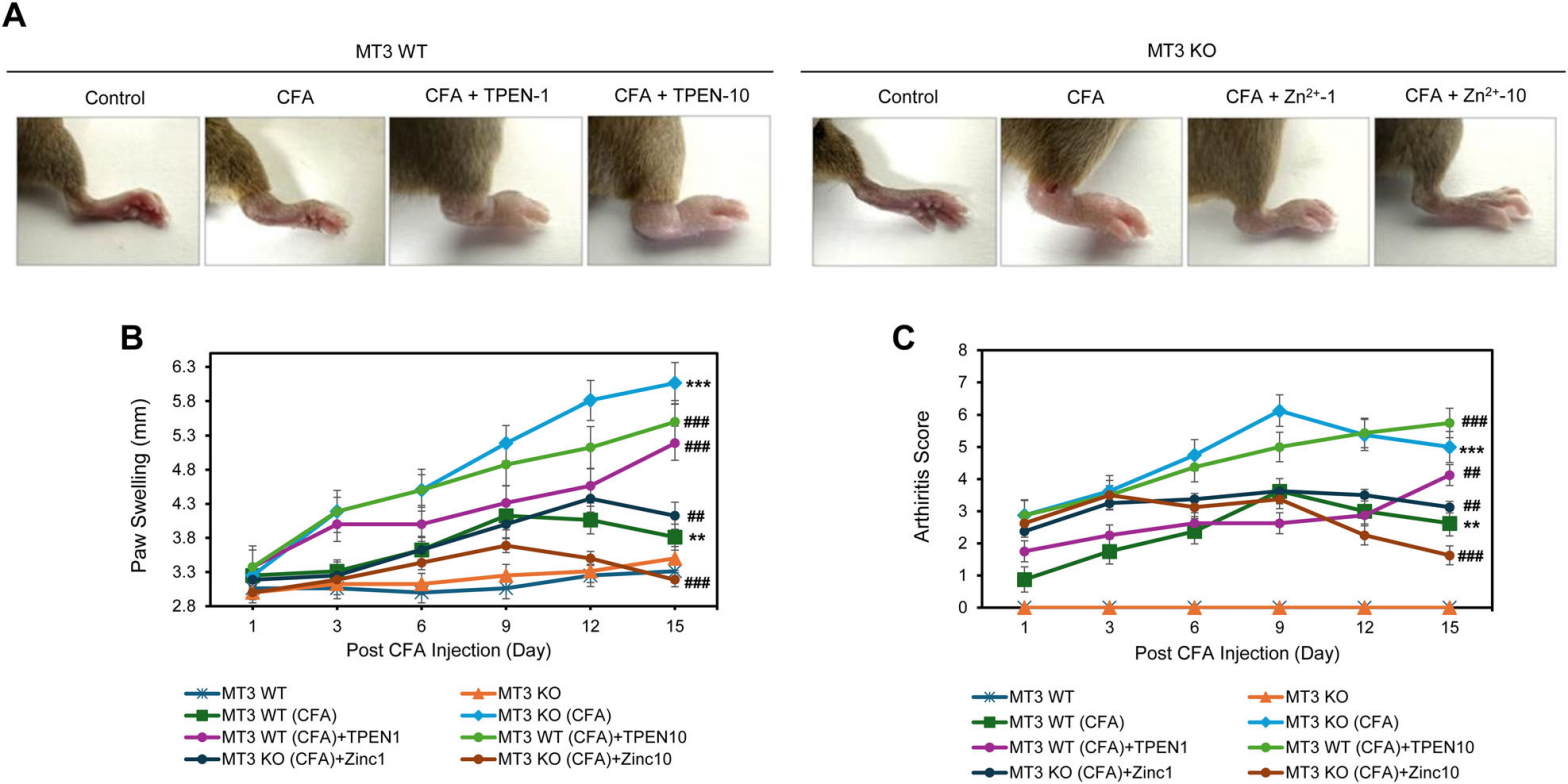
Involvement of Zn^2+^ and *MT3* in paw swelling and arthritis score in complete Freund’s adjuvant (CFA)-induced inflammatory pain mouse model. **A** Assessment of paw edema and swelling in treatment groups of *MT3* WT and KO mice, following treatment with various drug combinations. **B** Paw edema measurements recorded on days 0, 3, 6, 9, 12, and 15, expressed as foot breadth of the ankle using a Vernier caliper. **C** Arthritis index was calculated on days 0, 3, 6, 9, 12, and 15 as the mean value of the left and right hind paws in each group. The mice were assigned to the following eight experimental groups: (1) *MT3* WT (control group) (*n* = 8) receiving 0.9% saline and 2% dimethyl sulfoxide (DMSO), (2) *MT3* KO (control group) (*n* = 8), (3) *MT3* WT + 10 mg/mL CFA (*n* = 8), (4) *MT3* KO + 10 mg/mL CFA (*n* = 8), (5) *MT3* WT + 10 mg/mL CFA + 1 mg/kg TPEN (*n* = 8), (6) *MT3* WT + 10 mg/mL CFA + 10 mg/kg TPEN (*n* = 8), (7) *MT3* KO + 10 mg/mL CFA + 1 mg/kg ZnCl_2_ (*n* = 8), (8) *MT3* KO + 10 mg/mL CFA + 10 mg/kg ZnCl_2_ (*n* = 8). Mean (*n* = 8) ± SD. *P < 0.05, **P < 0.01, ***P < 0.001 compared to the control group; ^#^*P* < 0.05, ^##^*P* < 0.01, ^###^*P* < 0.001 compared to CFA-treated group; *ns*, not significant.

To investigate the effects of CFA, TPEN (an intracellular zinc chelator), and ZnCl_2_ on inflammatory pain progression, we recorded paw swelling and arthritis indices after combination drug treatments. The *MT3* KO control group exhibited greater paw swelling than did the *MT3* WT control group, with significant differences observed from days 3 to 15 (Fig. 2A, B). Paw swelling was increased in the CFA-treated *MT3* WT group compared with that in the untreated *MT3* WT control group (Fig. 2A, B). The *MT3* KO group treated with CFA demonstrated significantly more paw swelling than did the *MT3* WT group receiving the same treatment (Fig. 2A, B). Furthermore, in the *MT3* group treated with CFA, addition of 1 or 10 mg/kg TPEN elicited a marked increase in paw swelling compared to that in the *MT3* WT + CFA group without TPEN, revealing a TPEN concentration-dependent effect (Fig. 2A, B). Additional treatment with 1 or 10 mg/kg ZnCl_2_ in the *MT3* KO + CFA group mitigated paw swelling in a ZnCl_2_ concentration-dependent manner, reducing swelling to levels similar to those in the *MT3* KO control (Fig. 2A, B).

Moreover, arthritis scores were evaluated across the experimental groups from days 1 to 15. The findings revealed no significant differences in arthritis scores between the *MT3* KO and *MT3* WT control groups throughout the study period (Fig. 2C). The *MT3* WT + CFA group displayed an increase in arthritis scores compared with those in the non-CFA control group, whereas the *MT3* KO + CFA group showed a substantial increase in arthritis scores relative to those in the *MT3* WT + CFA group (Fig. 2C). Additionally, the *MT3* + CFA group, treated with either 1 or 10 mg/kg TPEN, demonstrated a notable increase in arthritis scores compared to scores in the *MT3* WT + CFA group in a TPEN concentration-dependent manner (Fig. 2C). Notably, treatment with 1 or 10 mg/kg ZnCl_2_ in the MT3 KO + CFA group significantly restored arthritis scores, reflecting Zn^2+^ concentration-dependent recovery (Fig. 2C).

### Zn^2+^ and *MT3* mitigate sensorimotor deficits in a CFA-induced inflammatory pain mouse model

**Fig. 3:**
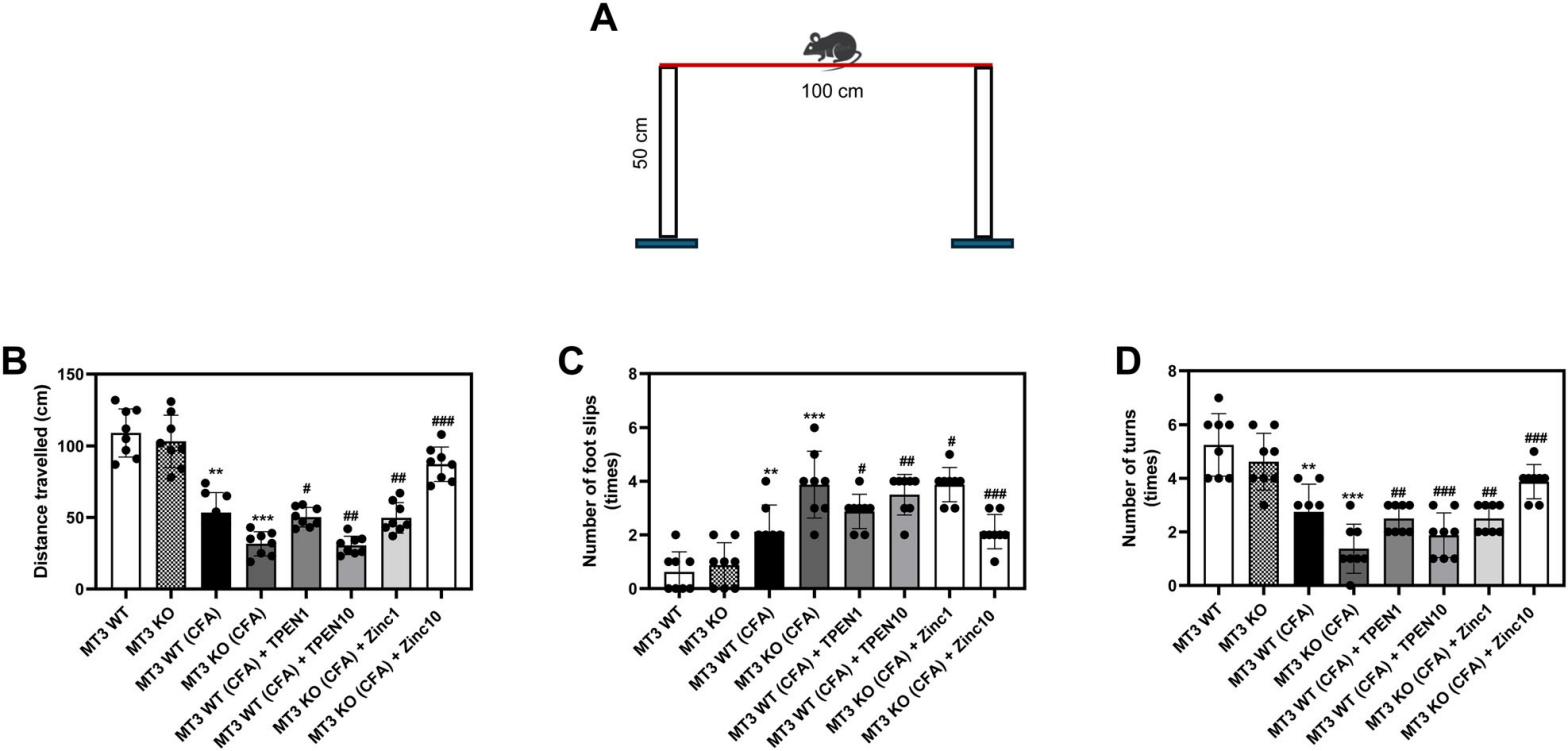
Effects of Zn^2+^ and *MT3* on sensorimotor deficits in complete Freund’s adjuvant (CFA)-induced inflammatory pain mouse model. **A** Schematic description of the experiment. **B** Total distance traveled, **C** Number of foot slips, and **D** Number of turns as measured by the walk beam test (WBT). Mice were divided into the following eight groups: (1) *MT3* WT (control group) (*n* = 8) received 0.9% saline and 2% dimethyl sulfoxide (DMSO), (2) *MT3* KO (control group) (*n* = 8), (3) *MT3* WT + 10 mg/mL CFA (*n* = 8), (4) *MT3* KO + 10 mg/mL CFA (*n* = 8), (5) *MT3* WT + 10 mg/mL CFA + 1 mg/kg TPEN (*n* = 8), (6) *MT3* WT + 10 mg/mL CFA + 10 mg/kg TPEN (*n* = 8), (7) *MT3* KO + 10 mg/mL CFA + 1 mg/kg ZnCl_2_ (*n* = 8), (8) *MT3* KO + 10 mg/mL CFA + 10 mg/kg ZnCl_2_ (*n* = 8). Results are presented as the mean (*n* = 8) ± SD. *P < 0.05, **P < 0.01, ***P < 0.001 compared to control group; ^#^*P* < 0.05, ^##^*P* < 0.01, ^###^*P* < 0.001 compared to CFA-treated group; *ns*, not significant.

**Fig. 4:**
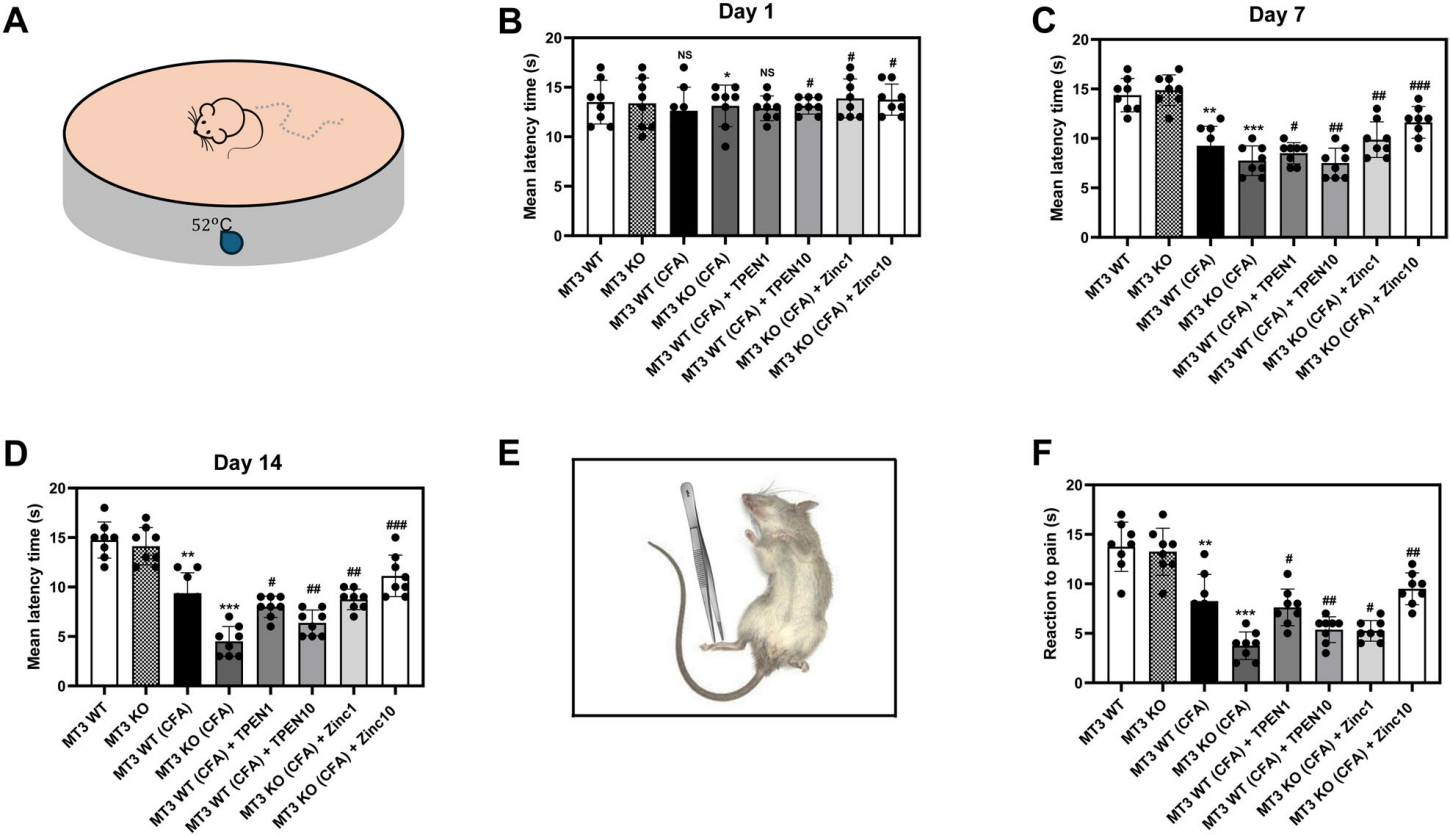
Effects of Zn^2+^ and *MT3* on antinociceptive activity and mechanical hyperalgesia in complete Freund’s adjuvant (CFA)-induced inflammatory pain. **A** Schematic representation of the behavioral test designed to measure mean latency time. **B**–**D** Mean latency times on days 1 (**B**), 7 (**C**), and 14 (**D**), assessed using the hot plate test. This test was conducted once per week to evaluate the degree of pain in the hind paws of each group. **E** Schematic overview of the mechanical hyperalgesia test. **F** Quantitative measurement of the nociceptive responses to pain. The nociceptive response to the application of a clip on the inflamed hind paw in CFA mice, serving as a model for mechanical hyperalgesia, was evaluated. Mice were divided into the following eight groups: (1) *MT3* WT (control group) (*n* = 8) received 0.9% saline and 2% dimethyl sulfoxide (DMSO), (2) *MT3* KO (control group) (*n* = 8), (3) *MT3* WT + 10 mg/mL CFA (*n* = 8), (4) *MT3* KO + 10 mg/mL CFA (*n* = 8), (5) *MT3* WT + 10 mg/mL CFA + 1 mg/kg TPEN (*n* = 8), (6) *MT3* WT + 10 mg/mL CFA + 10 mg/kg TPEN (*n* = 8), (7) *MT3* KO + 10 mg/mL CFA + 1 mg/kg ZnCl_2_ (*n* = 8), (8) *MT3* KO + 10 mg/mL CFA + 10 mg/kg ZnCl_2_ (n = 8). Results are presented as the mean (*n* = 8) ± SD. *P < 0.05, **P < 0.01, ***P < 0.001 compared to control group; ^#^*P* < 0.05, ^##^*P* < 0.01, ^###^*P* < 0.001 compared to CFA-treated group; *ns*, not significant.

Sensorimotor deficits in different groups of mice were evaluated using the walk beam paradigm (Fig. 3A). The findings indicated no significant differences between the *MT3* WT and KO control groups regarding the distance traveled, number of foot slips, or number of turns (Fig. 3B–D). In the *MT3* WT + CFA group, a reduction in the distance traveled and number of turns, coupled with an increase in foot slips, was observed compared to in the *MT3* WT control group (Fig. 3B–D). The *MT3* KO + CFA group exhibited a pronounced decline in the distance traveled and number of turns, along with a significant increase in foot slips, relative to the *MT3* WT + CFA group (Fig. 3B–D). Furthermore, *MT3* + CFA mice treated with 1 and 10 mg/kg TPEN demonstrated a concentration-dependent decrease in the distance and number of turns, accompanied by an increase in foot slips, compared to the untreated *MT3* WT + CFA group (Fig. 4B, D). Administration of Zn^2+^ at 1 or 10 mg/kg in *MT3* KO + CFA mice led to significant recovery, marked by an increase in distance traveled, a higher number of turns, and a substantial reduction in foot slips in a concentration-dependent manner compared to the *MT3* KO + CFA group without Zn^2+^ treatment (Fig. 3B–D).

### Zn^2+^ and *MT3* modulate antinociceptive activity in CFA-induced inflammatory pain in mice

Antinociceptive activity among the various groups was assessed on days 1, 7, and 14 (Fig. 4A–D). The different groups showed no significant differences on day 1 (Fig. 4B) but exhibited notable differences by day 7. Specifically, there was no significant difference in the pain threshold between the *MT3* WT and KO control groups (Fig. 4C). The *MT3* WT + CFA group exhibited a reduced pain threshold compared to the *MT3* WT control group, whereas the *MT3* KO + CFA group displayed a significantly diminished pain threshold relative to the *MT3* WT + CFA group (Fig. 4C).

Additionally, *MT3* + CFA mice treated with 1 or 10 mg/kg TPEN experienced a concentration-dependent reduction in the pain threshold compared to the untreated *MT3* WT + CFA group (Fig. 4C). In contrast, *MT3* KO + CFA mice receiving 1 or 10 mg/kg Zn^2+^ demonstrated prolonged pain threshold durations and significant antinociceptive effects as compared to the untreated *MT3* KO + CFA group, in a concentration-dependent manner (Fig. 4C). By day 14, similar results were observed as on day 17, although a trend toward exacerbated lesion activity was noted in pain threshold evaluations (Fig. 4D).

### Zn^2+^ and *MT3* mitigate CFA-induced mechanical hyperalgesia in mice

Mechanical hyperalgesia was assessed across the groups of experimental mice, as illustrated in Fig. 4E. The findings revealed significant variations in the latency to nociceptive behavior among the treatment groups. Specifically, no significant differences were observed in the pain threshold between the *MT3* WT and KO control groups in inflamed paws (Fig. 4F). In the *MT3* WT + CFA group, the pain threshold in the inflamed paw was reduced compared to that in the *MT3* WT control group. The *MT3* KO + CFA group exhibited a marked decrease in the pain threshold relative to the *MT3* WT + CFA group (Fig. 4F). Furthermore, *MT3* + CFA mice treated with 1 or 10 mg/kg TPEN showed a concentration-dependent reduction in the pain threshold in the inflamed paw compared to that in the untreated *MT3* WT + CFA group (Fig. 4F). In contrast, the *MT3* KO + CFA group administered 1 or 10 mg/kg Zn^2+^ showed enhanced recovery of the pain threshold in the inflamed paw, indicating a concentration-dependent improvement compared to the untreated *MT3* KO + CFA group (Fig. 4F).

### Zn^2+^ and *MT3* modulate immune organ indices in a CFA-induced inflammatory pain mouse model

**Fig. 5:**
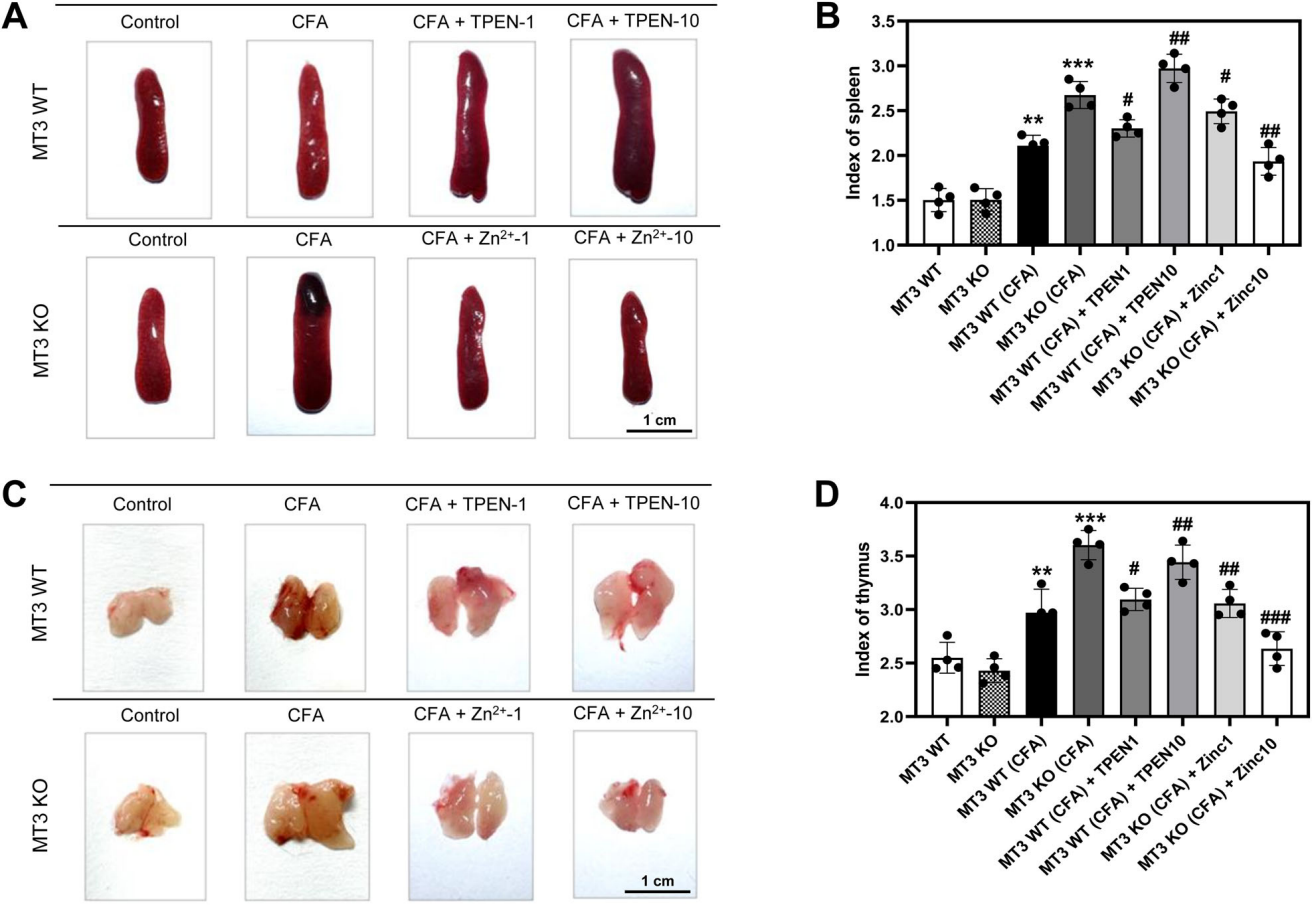
Protective role of Zn^2+^ and *MT3* on spleen and thymus indices in complete Freund’s adjuvant (CFA)-induced inflammatory pain in mice. **A** Abnormal enlargement of the spleen due to edema and swelling in the different treatment groups. **B** Spleen index measurements in the treatment groups. **C** Abnormal enlargement of the thymus due to edema and swelling in the treatment groups. **D** Thymus index measurements in the treatment groups. Mice were divided into eight experimental groups as follows: (1) *MT3* WT (control group) (*n* = 8) received 0.9% saline and 2% dimethyl sulfoxide (DMSO), (2) *MT3* KO (control group) (*n* = 8), (3) *MT3* WT + 10 mg/mL CFA (n = 8), (4) *MT3* KO + 10 mg/mL CFA (*n* = 8), (5) *MT3* WT + 10 mg/mL CFA + 1 mg/kg TPEN (*n* = 8), (6) *MT3* WT + 10 mg/mL CFA + 10 mg/kg TPEN (*n* = 8), (7) *MT3* KO + 10 mg/mL CFA + 1 mg/kg ZnCl_2_ (*n* = 8), (8) *MT3* KO + 10 mg/mL CFA + 10 mg/kg ZnCl_2_ (*n* = 8). Results are presented as mean (*n* = 8) ± SD. *P < 0.05, **P < 0.01, ***P < 0.001 compared to control group; ^#^*P* < 0.05, ^##^*P* < 0.01, ^###^*P* < 0.001 compared to CFA-treated group; *ns*, not significant.

Inflammatory pain in the paw like arthritis affects immune organs, such as the spleen and thymus. To explore whether Zn^2+^ and *MT3* contributed to CFA-induced abnormalities in immune organ indices, we examined their effects on the spleen and thymus. The indices of these organs did not significantly differ between the *MT3* WT and *MT3* KO control groups (Fig. 5). In the *MT3* WT + CFA group, the thymus and spleen indices were elevated compared to those in the *MT3* WT control group (Fig. 5). Similarly, the *MT3* KO + CFA group exhibited a significant increase in these indices compared to the *MT3* WT + CFA group (Fig. 5). Additionally, *MT3* + CFA group treated with TPEN at doses of 1 and 10 mg/kg demonstrated a concentration-dependent increase in thymus and spleen indices relative to the *MT3* WT + CFA group treated with TPEN (Fig. 5). Furthermore, Zn^2+^ administration at 1 and 10 mg/kg to the *MT3* KO + CFA group significantly reduced the thymus and spleen indices, showing concentration-dependent amelioration compared to the *MT3* KO + CFA group without Zn^2+^ treatment (Fig. 5).

### Association between CFA-induced inflammatory pain severity and alterations in Zn^2+^ and *MT3* levels

**Fig. 6:**
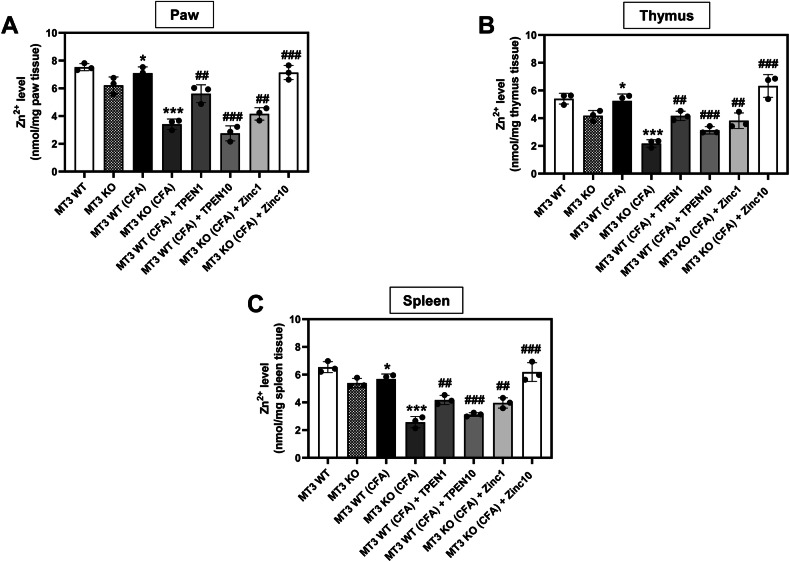
Changes in Zn^2+^ concentration in complete Freund’s adjuvant (CFA)-induced inflammatory pain in *MT3* knockout mouse model. The Zn^2+^ concentration changes in the **A** paw, **B** thymus, and **C** spleen of *MT3* knockout mice with CFA-induced inflammatory pain. Zn^2+^ levels were measured using a kit. Mice were divided into the following eight experimental groups: (1) *MT3* WT (control group) (*n* = 8) received 0.9% saline and 2% dimethyl sulfoxide (DMSO), (2) *MT3* KO (control group) (*n* = 8), (3) *MT3* WT + 10 mg/mL CFA (*n* = 8), (4) *MT3* KO + 10 mg/mL CFA (*n* = 8), (5) *MT3* WT + 10 mg/mL CFA + 1 mg/kg TPEN (*n* = 8), (6) *MT3* WT + 10 mg/mL CFA + 10 mg/kg TPEN (*n* = 8), (7) *MT3* KO + 10 mg/mL CFA + 1 mg/kg ZnCl_2_ (*n* = 8), (8) *MT3* KO + 10 mg/mL CFA + 10 mg/kg ZnCl_2_ (*n* = 8). Results are presented as the mean (*n* = 8) ± SD. *P < 0.05, **P < 0.01, ***P < 0.001 compared to control group; ^#^*P* < 0.05, ^##^*P* < 0.01, ^###^*P* < 0.001 compared to CFA-treated group; *ns*, not significant.

To examine whether the zinc concentration is closely linked to inflammatory pain severity, we quantified the total Zn^2+^ levels in the paw, thymus, and spleen tissues. As shown in Fig. 6, although there were no significant differences, the *MT3* KO control group exhibited slightly lower Zn^2+^ concentrations in the paw, thymus, and spleen than the *MT3* WT control group. In the *MT3* WT + CFA group, total Zn^2+^ levels in these tissues were markedly reduced relative to those in the *MT3* WT control group (Fig. 6A–C). Furthermore, the *MT3* WT group treated with TPEN at 1 or 10 mg/kg showed a significant decrease in the total Zn^2+^ content in the paw, thymus, and spleen compared to that in the untreated *MT3* WT + CFA group in a TPEN concentration-dependent manner (Fig. 6A–C). *MT3* KO + CFA groups receiving ZnCl_2_ at either 1 or 10 mg/kg showed significant restoration of Zn^2+^ levels in these tissues compared to the *MT3* KO + CFA group without ZnCl_2_, with changes corresponding to the ZnCl_2_ concentration (Fig. 6A–C).

### Oxidative stress and Zn^2+^ and *MT3* regulation of enzymatic antioxidants in CFA-induced inflammatory pain in mice

Oxidative stress, a primary consequence of cellular toxicity, leads to cell, DNA, and tissue damage and promotes severe inflammatory responses when persistent. To assess the protective role of *MT3* in regulating intracellular Zn^2+^ activity in CFA-induced inflammatory pain, we evaluated antioxidant enzyme activities in the paw and spleen of *MT3* knockout mice.

**Fig. 7:**
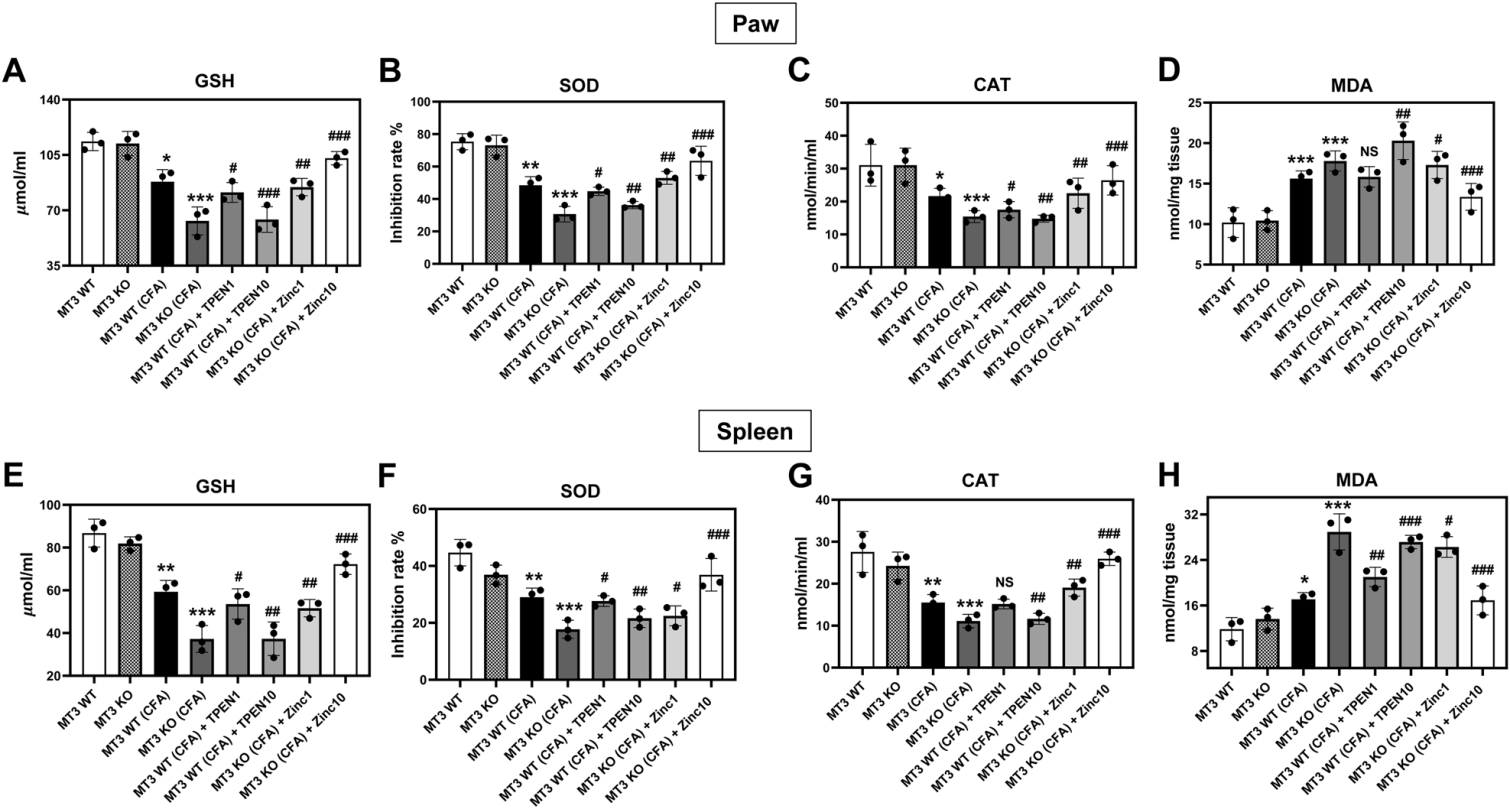
Effects of Zn^2+^ and *MT3* on the activities of antioxidant-associated molecules in complete Freund’s adjuvant (CFA)-induced inflammatory pain mice. Significant changes in **A**, **E** glutathione (GSH), **B**, **F** superoxide dismutase (SOD), **C**, **G** catalase (CAT), and **D**, **H** malondialdehyde (MDA) in the paw (**A**–**D**) and spleen (**E**–**H**) tissues. Mice were divided into eight experimental groups as follows: (1) *MT3* WT (control group) (*n* = 8) received 0.9% saline and 2% dimethyl sulfoxide (DMSO), (2) *MT3* KO (control group) (*n* = 8), (3) *MT3* WT + 10 mg/mL CFA (*n* = 8), (4) *MT3* KO + 10 mg/mL CFA (*n* = 8), (5) *MT3* WT + 10 mg/mL CFA + 1 mg/kg TPEN (*n* = 8), (6) *MT3* WT + 10 mg/mL CFA + 10 mg/kg TPEN (n = 8), (7) *MT3* KO + 10 mg/mL CFA + 1 mg/kg ZnCl_2_ (*n* = 8), (8) *MT3* KO + 10 mg/mL CFA + 10 mg/kg ZnCl_2_ (*n* = 8). Results are presented as the mean (*n* = 8) ± SD. *P < 0.05, **P < 0.01, ***P < 0.001 compared to control group; ^#^*P* < 0.05, ^##^*P* < 0.01, ^###^*P* < 0.001 compared to CFA-treated group; *ns*, not significant.

There were no significant differences in the activities of GSH, SOD, CAT, and MDA between the *MT3* KO and *MT3* WT control groups (Fig. 7). However, in the *MT3* WT + CFA group, a decrease in GSH, SOD, and CAT activities and an increase in MDA levels were observed in the paw and spleen compared to those in the non-CFA control group (Fig. 7). In the *MT3* KO + CFA group, there was a notable reduction in GSH, SOD, and CAT activities, accompanied by elevated MDA levels in the paw and thymus compared to those in the *MT3* WT + CFA group (Fig. 7). Furthermore, the *MT3* + CFA group treated with TPEN at either 1 or 10 mg/kg exhibited a significant reduction in GSH, SOD, and CAT activities, along with an increase in MDA levels in the paw and thymus relative to those in the untreated *MT3* WT + CFA group in a TPEN concentration-dependent manner (Fig. 7).

Treatment of the *MT3* KO + CFA group with ZnCl_2_ at 1 or 10 mg/kg significantly enhanced GSH, SOD, and CAT activities and reduced MDA levels compared to those in the *MT3* KO + CFA group without ZnCl_2_ in a ZnCl_2_ concentration-dependent manner (Fig. 7).

### Zn^2+^ and *MT3* modulate cytokine-driven inflammation in RA mouse model

**Fig. 8:**
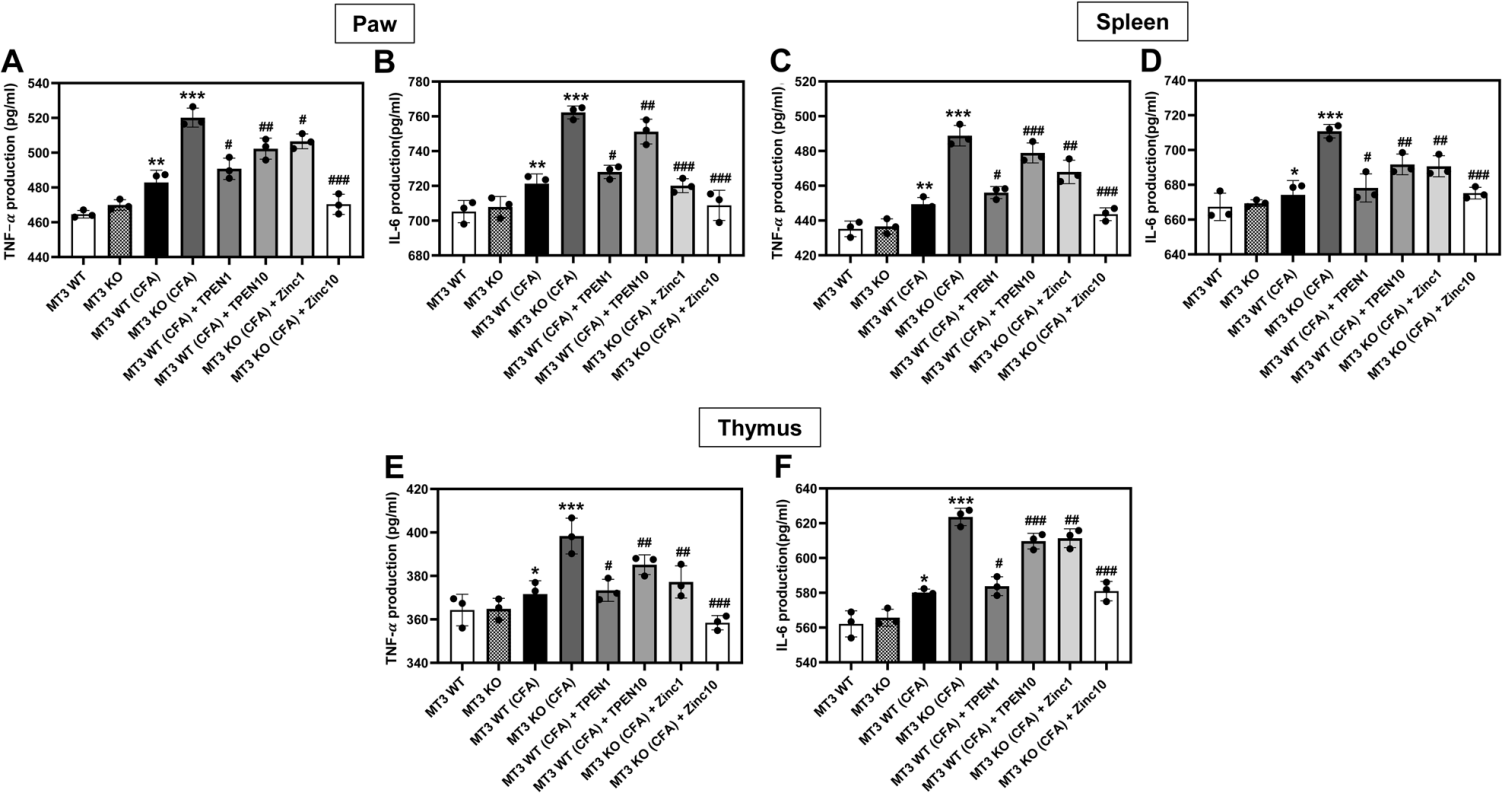
Role of Zn^2+^ and *MT3* on cytokine levels in the paw, spleen, and thymus in complete Freund’s adjuvant (CFA)-induced inflammatory pain in mice. Levels of cytokines, TNF-$\alpha$ and IL-6 in **A**, **B** the paw, **C**, **D** spleen, and **E**, **F** the thymus tissues of inflammatory pain mice induced by CFA. Mice were divided into eight experimental groups as follows: (1) *MT3* WT (control group) (*n* = 8) received 0.9% saline and 2% dimethyl sulfoxide (DMSO), (2) *MT3* KO (control group) (*n* = 8), (3) *MT3* WT + 10 mg/mL CFA (*n* = 8), (4) *MT3* KO + 10 mg/mL CFA (*n* = 8), (5) *MT3* WT + 10 mg/mL CFA + 1 mg/kg TPEN (*n* = 8), (6) *MT3* WT + 10 mg/mL CFA + 10 mg/kg TPEN (*n* = 8), (7) *MT3* KO + 10 mg/mL CFA + 1 mg/kg ZnCl_2_ (*n* = 8), (8) *MT3* KO + 10 mg/mL CFA + 10 mg/kg ZnCl_2_ (*n* = 8). Results are presented as the mean (*n* = 8) ± SD. *P < 0.05, **P < 0.01, ***P < 0.001 compared to control group; ^#^*P* < 0.05, ^##^*P* < 0.01, ^###^*P* < 0^.^001 compared to CFA-treated group; *ns*, not significant.

To investigate the regulatory roles of Zn^2+^ and *MT3* in CFA-induced inflammatory processes, we conducted cytokine assays targeting TNF-α and IL-6 in the paw, spleen, and thymus of mice. The findings revealed no significant differences in TNF-α and IL-6 levels across these tissues between the *MT3* WT and *MT3* KO control groups (Fig. 8). However, in the *MT3* WT + CFA group, TNF-α and IL-6 levels in the paw, spleen, and thymus were notably elevated compared to those in the control group not exposed to CFA (Fig. 8). Furthermore, the *MT3* KO + CFA group exhibited a marked increase in TNF-α and IL-6 levels across these tissues in comparison to the *MT3* WT + CFA group (Fig. 8). In addition, *MT3* + CFA groups treated with TPEN at 1 or 10 mg/kg showed a significant, TPEN concentration-dependent increase in TNF-$\alpha$ and IL-6 levels in the paw, spleen, and thymus tissues compared to the *MT3* WT + CFA group untreated with TPEN (Fig. 8). Moreover, treatment of the *MT3* KO + CFA group with Zn^2+^ at 1 or 10 mg/kg yielded potent inhibitory effects on TNF-α and IL-6 production in the same tissues, demonstrating a Zn^2+^ concentration-dependent suppression when compared to the *MT3* KO + CFA group untreated with Zn^2+^ (Fig. 8).

## Discussion

As a chronic autoimmune condition, inflammatory pain in paw is characterized by cytokine-driven inflammation of the synovial membrane, ultimately leading to cartilage degradation, bone erosion, and joint deformities [32]. The clinical manifestations of inflammatory pain in paw frequently include pain, stiffness, swelling, deformity, and progressive loss of joint function [33].

Our findings demonstrate that *MT3* deficiency exacerbates inflammatory and pain responses induced by subcutaneous CFA injections in mice. *MT3* deficiency worsened sensorimotor deficits in the walking beam test following CFA administration. Furthermore, *MT3* deficiency intensified CFA-induced anxiety- and depression-like behaviors. Biochemical analyses revealed that *MT3* deficiency resulted in elevated oxidative stress within the paw and spleen along with a reduction in Zn^2+^ levels in CFA-induced inflammatory pain mice. Moreover, *MT3* deficiency was associated with increased CFA-induced IL-6 and TNF-α levels in the paw, spleen, and thymus. *MT3*-deficient mice exhibited a pronounced reduction in body mass and significantly elevated arthritis scores compared to those in the control groups.

Previous studies showed that inflammatory pain impairs physical functioning including motor difficulties and functional sensorimotor impairments [34]. Zn^2+^ supplementation reversed dysfunctional locomotor activity in CFA-induced inflammatory pain in *MT3* KO mice. We showed that CFA substantially impaired physical motor function, as evidenced by decreased mobility, increased fall incidence, and reduced rotational movements in the walking beam test. The alleviation of CFA-induced sensorimotor deficits with increased intracellular Zn^2+^ levels suggests that Zn^2+^ can enhance the quality of life of patients with inflammatory pain.

The thymus and spleen are integral components of the immune system, and their relative weights are often used as preliminary indices to assess the immunomodulatory effects of a drug. The spleen serves a crucial function by filtering and eliminating senescent and damaged erythrocytes, as well as removing antigens through phagocytosis, thus bolstering immunity. This activity is vital for infection prevention, as it promotes the production of lymphocytes, which are pivotal in the body’s initial defense against invading pathogens [35]. Similarly, the thymus plays a central role in the immune system by processing immature T lymphocyte precursors from the bone marrow, initially localized within the outer cortex of the thymus, into functionally competent T cells, which are crucial for adaptive immunity [36]. Our findings demonstrate that increasing Zn^2+^ levels effectively reverses the enlargement of both the spleen and thymus, which is otherwise induced by *MT3* deficiency in a CFA-induced inflammatory pain model. This result underscores the important role of Zn^2+^ in immune regulation, primarily through inhibition of splenic and thymic hypertrophy and broader systemic proliferation of immune cells.

CFA-induced inflammatory pain in the paw rodents mimics the clinical and pathological characteristics of arthritis, making it the most prevalent model for investigating disease etiology, mechanisms, and development of anti-inflammatory pain agents [37, 38]. Previous studies established that unilateral dermal injection of CFA induces a robust inflammatory response in rodents [39]. This inflammation is mediated by chemical factors such as IL-6, IL-1, and TNF-α, which play pivotal roles in the progression of inflammatory arthritis by facilitating the binding and action of other inflammatory agents, including prostaglandins, nitric oxide, matrix metalloproteinase, and C-reactive protein [40, 41]. Specifically, IL-6 has been implicated in the adhesion molecule upregulation, osteoclast activation, and B cell activation [42, 43]. Increased expression of adhesion molecules on endothelial cell walls promotes an influx of inflammatory cells into the active joints, leading to the release of cytotoxic metabolites, such as reactive oxygen species. In this context, our findings indicate that Zn^2+^ supplementation significantly attenuates CFA-induced inflammatory edema in *MT3*-deficient mice, suggesting that *MT3* can confer beneficial effects in inflammatory pain management. Moreover, Zn^2+^ enhancement markedly reduced the levels of IL-6 and TNF-α, two principal cytokines that initiate and propagate the inflammatory process. This inhibition of inflammatory cytokine release further implies that Zn^2+^ modulates cytokine levels, thereby contributing to its potential therapeutic role in controlling arthritis-associated inflammatory responses. Application of antioxidants has been proposed as a promising strategy for the prevention and treatment of chronic inflammatory diseases [44, 45]. Our findings revealed that Zn^2+^ supplementation led to potent antioxidant effects, as evidenced by a reduction in MDA levels and an increase in antioxidant molecules, namely GSH, SOD, and CAT in the paw tissue and spleen of CFA-induced MT3 knockout mice.

We demonstrated the role of *MT3* in an *MT3* knockout mouse model of inflammatory pain in the paw, highlighting the necessity of intracellular Zn^2+^ and its interplay with *MT3* in mitigating pain- and depression-related behaviors. Additionally, these findings underscore the significance of *MT3* in attenuating the inflammatory pain processes influenced by oxidative stress and inflammatory cytokines. Further research is needed to determine whether *MT3*’s molecular mechanisms directly involve interactions with key immune targets, thereby contributing to the alleviation of CFA-induced inflammatory pain in a clinical context.

## Materials and methods

### Chemicals and antibodies

CFA (Cat#F5881), N,N,N′,N′-Tetrakis(2-pyridylmethyl)ethylenediamine (TPEN) (Cat#P4413), zinc chloride (ZnCl_2_) (Cat#Z0152), potassium acetate (#127-08-2), and chloroform (#67-66-3) were from Sigma-Aldrich (St. Louis, MO, USA). The EnzyChrom^TM^ GSH/GSSG Assay Kit (Cat#EGTT-100) and QuantiChrom #Zinc Assay Kit (Cat#DIZN-250) were obtained from BioAssay Systems (Hayward, CA, USA). Assay kits for SOD (Cat#K335-100) and CAT activity (colorimetric/fluorometric) (Cat#K773-100) were acquired from BioVision (Waltham, MA, USA). The mouse TNF-α ELISA kit (Cat#CSB-E04741m) and mouse IL-6 ELISA kit (Cat#CSB-E04639m) were from Cusabio Technology (Huston, TX, USA). AccuPower® PCR PreMix (Cat#K-2012) and DNA Ladder reagents were purchased from BioNEER (Daejeon, Korea). *MT3*-WT (Cat#OLA496390-001), *MT3*-C (Cat#OLA496390-002), and *MT3*-KO primers (Cat#OLA496390-003) were custom-designed by Cosmo Genetech (Seoul, Korea). Pierce^TM^ RIPA buffer (Cat#89900) and recombinant PCR-grade proteinase K (Cat#EO0491) were obtained from Thermo Scientific (Waltham, MA, USA).

### Establishment of CFA-induced inflammatory pain mouse model

#### Animals

Eight-week-old male 129S2/SvPasCrl (129-Elite (SOPF) mouse) strain, 8-week-old female *MT3* KO strain (Charles River Laboratories, Wilmington, MA, USA), and 8-week-old female C57BL/6N strain (Samtaco, Suwon, Korea) were used to generate the *MT3* background mouse model. SV129 male and C57BL/6N female mice were crossbred to establish an SVC strain. Subsequently, SVC males were matched with *MT3* KO females to produce an *MT3* heterozygous (hetero) strain. Further breeding of male and female *MT3* hetero mice resulted in three genotypes: *MT3* WT, *MT3* KO, and *MT3* hetero.

The mice were housed in a controlled environment with unrestricted access to water and standard rodent chow, under conditions of 23 ± 3 °C temperature, 55% ± 10% humidity, and a 12-h light/dark cycle. Stringent measures were taken to minimize the use of animals and alleviate any potential suffering. Evaluations and records of behavioral test results were evaluated by a blind person throughout the experimental process. All experimental protocols were approved by the Animal Experimentation Ethics Committee of Jeonbuk National University (Approval Number: JBNU 2024-0346).

#### Genotyping

Offspring from the cross between *MT3* hetero females and males were genotyped to identify *MT3* WT, *MT3* KO, and *MT3* hetero strains. A 0.5 cm tail segment was collected, to which lysis buffer and proteinase K were added; this sample was incubated overnight at 56 °C. Potassium acetate (5 M) was added, and the mixture was incubated on ice for 3 min, followed by addition of chloroform, shaking, and incubation on ice for 15 min. The samples were centrifuged at 12,000 rpm, 4 °C for 20 min. The supernatant was collected, mixed with 100% ethanol, and centrifuged. This procedure was repeated using 70% ethanol, and the supernatant was removed, allowing the sample to air-dry at room temperature. This process yielded purified DNA that was dissolved in distilled water for further analysis.

Polymerase chain reaction (PCR) was performed using a TaKaRa Thermal Cycler Dice Touch (Shiga, Japan) according to the following program: pre-denaturation at 94 °C for 3 min; 40 cycles for *MT3* with denaturation at 94 °C for 30 s, annealing at 63 °C for 1 min, and extension at 72 °C for 1 min; followed by final extension at 70 °C for 3 min. The reaction mixture was maintained at 4 °C indefinitely. The primer sequences for PCR were: *MT3* WT: 5′-CCT AGC ACC CAC CCA AAG AGC TG-3′; *MT3* KO: 5′-GGC TCT ATG GCT TCT GAG GCG G-3′; *MT3* C: 5′-GGT CCT CAC TGG CAG CAG CTG C-3′. After PCR, the samples were separated by electrophoresis using a Mupid-2 Plus system (Optima, Inc., Tokyo, Japan), and the results were confirmed. Electrophoresis identified the genotypes of *MT3* WT, *MT3* KO, and *MT3* hetero mice, as shown in Fig. 1A. Newly identified 8-week-old male *MT3* WT and *MT3* KO mice from genotyping analysis were used in the inflammatory pain model experiment.

#### Establishment of inflammatory pain mouse model

The mice were divided into eight experimental groups (*n* = 64) as follows: (1) *MT3* WT (control group) (*n* = 8) received 0.9% saline and 2% dimethyl sulfoxide (DMSO); (2) *MT3* KO (control group) (*n* = 8); (3) *MT3* WT + 10 mg/mL CFA (inflammatory pain) (*n* = 8); (4) *MT3* KO + 10 mg/mL CFA (inflammatory pain) (*n* = 8); (5) *MT3* WT + 10 mg/mL CFA + 1 mg/kg TPEN (*n* = 8); (6) *MT3* WT + 10 mg/mL CFA + 10 mg/kg TPEN (*n* = 8); (7) *MT3* KO + 10 mg/mL CFA + 1 mg/kg ZnCl_2_ (*n* = 8); (8) *MT3* KO + 10 mg/mL CFA + 10 mg/kg ZnCl_2_ (*n* = 8). For inflammatory pain groups (3) through (8), mice were immunized with a single dose of 0.1 mL CFA injected intradermally into the left hind metatarsal footpad on day 1, with the control groups (1) and (2) receiving no CFA. In addition, groups (5) and (6) received daily intraperitoneal injections of TPEN at doses of 1 and 10 mg/kg, respectively, whereas groups (7) and (8) received ZnCl_2_ at 1 and 10 mg/kg daily from day 1 to 14. The control group received an equivalent volume of water. All drug solutions were freshly prepared prior to administration. A schematic representation of the experimental timeline is shown in Fig. 1B.

#### Assessment of inflammatory pain in hind paw

##### Paw swelling

Paw edema was measured on days 0, 3, 6, 9, 12, and 15 using a Vernier caliper, with the results expressed as the foot breadth at the ankle. On day 15, mice were euthanized by decapitation. The paws, thymus, and spleens were carefully dissected, rinsed with ice-cold saline, blotted dry, and weighed [46]. The thymus and spleen indices were calculated as the ratio of the wet weight of these organs to the body weight (mg/g) of the mice, respectively [47].

##### Arthritis index score

The clinical severity of CFA-induced inflammatory pain in mice was evaluated using a standardized scoring system [48] as follows: 0, no signs of disease; 1, mild swelling and erythema of the ankle or toes; 2, moderate swelling and erythema of the ankle or toes; 3, severe swelling and erythema of the ankle or toes; and 4, deformation or stiffness of the ankle or toe joints. The cumulative arthritis score for each mouse reached a maximum of 8 (4 points per hind paw). The arthritis index for each group was determined by calculating the mean scores for the left and right hind paws.

### Behavior tests

#### Postural stability assessment

Sensorimotor function was evaluated in a walk beam test, which assesses fine motor coordination and balance in both naïve and drug-treated animals. The beam apparatus consisted of a 100 cm long, 2 cm wide wooden beam elevated to a height of 40 cm. The beam was coated with black paint and marked at 5 and 1 cm intervals for reference. The walk beam test comprises two phases: trial and test.

During the test phase, each mouse was placed at one end of the beam and allowed to freely traverse for 2 min after securing its grip. Between trials, the beam was cleaned with 70% ethanol and dried. During the test phase, the total distance traveled (cm), number of foot slips, and turning behaviors were recorded over a 2-min period. The total distance traveled was calculated by multiplying the number of times the animal crossed the beam by its final position on the beam. Foot slip was defined as any instance in which the animal’s hindfoot slipped off the beam. Turning behavior was recorded when the animal turned and proceeded in the opposite direction upon reaching the beam end.

#### Hot plate test

The hot plate test was conducted weekly from days 1 to 14 to assess the degree of pain in the hind paws of each group, as described by Sulaiman et al. [49]. The temperature of the hot plate was maintained at 52.0 ± 0.2 °C, and the soles of the mice were placed on the hot plate. The latency to a clear pain response such as paw withdrawal or licking (in seconds) was recorded. Each paw was tested in parallel, and the average time between the two measurements was recorded. If no pain response was observed within 20 s, the paw was immediately removed from the hot plate and the pain threshold was recorded at 20 s. The interval between measurements was at least 15 min.

#### Mechanical hyperalgesia test

The nociceptive response to mechanical hyperalgesia in CFA-treated mice was evaluated at 15 min post-CFA injection. The inflamed hind paws were subjected to mechanical stimulation by applying a clip, and the time (in seconds) taken for the animals to bite the clip, which is an indicator of nociception, was recorded. To prevent tissue damage, a cutoff time of 60 s was imposed, and the clip was immediately removed if the animal displayed signs of pain. Mice in the vehicle control group, which were not pretreated with CFA, were also subjected to pain assessment.

#### Immune organ index

On day 15 post-immunization, the mice were euthanized under anesthesia (pentobarbital sodium, 40 mg/kg, intraperitoneally). The thymus and spleen were immediately harvested and weighed. Indices for the spleen and thymus were calculated as the ratio of the wet weight of these organs to the body weight of the mice (mg/g) [47].

#### Determination of Zn^2+^ level in paw, thymus, and spleen

To measure Zn^2+^ levels in the tissues, 50–100 mg of frozen paw, thymus, and spleen tissues from a previously described reversibility study were thawed at room temperature, rinsed three times with cold PBS, and homogenized for 30 s at maximum speed in 1 mL of tissue protein extraction reagent (Pierce, Rockford, IL, USA) using a Polytron R PT 2100 homogenizer (Capitol Scientific, Austin, TX, USA). The homogenates were clarified by centrifugation at 10,000 × *g* for 15 min at 4 °C, and the supernatants were used for Zn^2+^ quantification. Zn^2+^ levels were measured using a QuantiChrom™ Zinc Assay kit (BioAssay Systems), following the manufacturer’s instructions.

### Oxidative stress measurement

#### Glutathione activity estimation

Reduced GSH levels in the paw and spleen on day 15 were quantified at an absorbance of 412 nm using a microplate reader (Epoch Microplate Spectrophotometer, Bio-Tek Instruments, Winooski, VT, USA). Measurements were conducted in accordance with the manufacturer’s protocols for commercially available assay kits, as previously described [50].

#### Antioxidant enzyme activity estimation

Antioxidant enzyme activity was measured according to the manufacturer’s instructions using commercially available assay kits [50, 51]. CAT activity was quantified by recording absorbance at 540 nm, whereas SOD activity was measured at 450 nm, using a microplate reader (Epoch™ Microplate Spectrophotometer).

#### MDA activity estimation

MDA levels in the paw and spleen on day 15 were quantified at an absorbance of 532 nm using a microplate reader (Epoch™ Microplate Spectrophotometer). The assay was performed according to the manufacturer’s instructions using a commercially available kit as previously reported [52].

### Inflammatory cytokine levels in paw, thymus, and spleen

TNF-α and IL-6 in the paw, spleen, and thymus tissues were determined after allowing the samples to clot for 1 h at room temperature. The tissues were collected and stored at −20 °C until further analysis. The concentrations of TNF-α and IL-6 in the tissues were quantified using enzyme-linked immunosorbent assay following the manufacturer’s protocol. All tissue samples were individually assayed for TNF-α and IL-6 using the respective test kits.

### Data analysis

All statistical analyses were performed using GraphPad Prism 7 software (San Diego, CA, USA). Data are expressed as the mean ± SEM. Comparisons between groups were performed using Student’s *t*-tests. Results are reported as not statistically significant unless otherwise indicated (*P < 0.05, **P < 0.01, ***P < 0.001 compared with control; ^#^*P* < 0.05, ^##^*P* < 0.01, ^###^*P* < 0.001 compared with treated CFA; *ns*, not significant; paired *t*-test).

## Data Availability

The data presented in this study are available on request from the corresponding author.
